# Give the College a Chance

**Published:** 1917-09-29

**Authors:** 


### NURSING AFFAIRS IN IRELAND.

### "GIVE THE COLLEGE A CHANCE."

I was interested to read a criticism of my remarks concerning the Irish Nursing Board in the *Poor-Law Officers' Journal.* I am a thorough believer in the beneficial effect of criticism, and especially of hostile criticism, provided that it is honest. The writer found difficulty in accepting my story as the whole truth, and exclaims, "There is evidently more at the bottom of this than is here explained." I am not surprised, for the story of a self-appointed Committee of Nurses establishing themselves by a provision in the by-laws for a six years' undisturbed tenure of office appears incredible. Nevertheless, this is true, and at the moment an endeavour is being made by some despairing supporters to obtain other nurses' signatures to a petition asking for amendment. Moreover, a prominent Dublin surgeon pointed out to the authors how monstrous is the provision, but without avail. These ladies hold the opinion of the rank and file of the profession in contempt, and entirely fail to realise that the committee of any association is a voice, and not an autocracy. The aim of the Irish Nursing Board is to govern a body of women whose sole function will consist of paying fees, and, so far, no revelation has been made as to what cause the fees will be devoted. Presumably they will be pooled and divided amongst themselves as remuneration for keeping a Register and conducting examinations. If this is a half-told tale, presumably the nursing press is open to receive a supplement.

### "A Damn Party in a Parlour."

Like many other Irish problems, the present condition of affairs in the nursing world here finds its origin in history, and although readers in Great Britain may experience difficulty in understanding the situation, to the initiated it is simplicity itself. The one and only Nurses' Association which existed here until quite recently, consisting of an inactive committee supported by a general body of mutes, was governed by a ring of matrons, in office or retired, all of whom had achieved their ambition in life, or, at all events, had climbed to the ultimate limit of their own particular ladders. Movements in the nursing world interested them as spectators, not as aspirants, and it was a matter of entertainment for them to meet together periodically to discuss the situation, whatsoever it might be, in philosophic fashion. The juniors throughout the country knew nothing of these proceedings, and probably cared nothing, but in either case they received no information concerning them. Without being disrespectful, discourteous, or flippant, may I suggest that these meetings corresponded with what Charles Lamb described as "a damn party in a parlour"? The obvious and natural result ensued. They formed into cliques, and the true explanation of the origin of the Irish Nursing Board is that the ladies who called it into being were sore, very sore, at the prospect of being left without portfolios when the College of Nursing established itself in Ireland. If it were a principle that was at stake, they never would have sought to borrow the programme of the College.

### The Most Direct Way to State Registration.

The writer in the *Poor-Law Officers' Journal,* in his concluding remarks, observes that nobody has as yet demonstrated what advantage either nurses or nursing will obtain either from the Irish Nursing Board or the College, and dismisses both with a wave of the hand and "a plague on both your houses." Is this wholesale attitude of dismissal not a little premature? So far as Ireland is concerned, I venture to suggest that it is. The College offers the most direct, if not the only, way to State Registration. It is extremely influentially backed, and its sole *raison d'être* is to benefit the profession. Its ultimate triumph must depend on the nurses themselves. The Irish branch has been scarcely established, and a sub-committee has already been appointed to inquire into and report on the economic question, to which I have been recently calling attention. A public meeting of nurses is to be summoned in Dublin in October, where there will be at least freedom of speech. I think I am accurate in stating that, except under the auspices of the College or in connection with its establishment, no public meeting of nurses was ever held in Dublin with the object of discussing the affairs of the profession.

### The College's Home Rule Principle.

A highly important branch of nursing in Ireland is that of the Poor Law, whose members have many substantial grievances. These nurses should unify and come within the fold of the College, and the College, on its part, will bid them welcome. As a matter of fact, many have already enrolled, and two members of the Board are actually Poor-Law matrons. The College of Nursing doubtless has its duties and responsibilities, but so likewise has the nurse. A feature of the College which should especially appeal to many Irish nurses is that it embraces the Home Rule principle. The Irish Board, in its miniature way, is a Nurses' Irish Parliament, and hence my remark that the nurse must take advantage of her opportunities if there is to be progress. The man in the street may abuse a shop without entering its doors, and all the time the goods he is in quest of may be on the counter. It is not therefore unreasonable to say, Give the College a chance.

### Yet Another Nursing Proposition.

During the past week a meeting of Irish country doctors was held at the Shelbourne Hotel, Dublin, with the primary object, apparently, of re-establishing the County Surgeons' Association, which had been for some time in suspense. The subject of nurses and their welfare occupied a good deal of attention, and some altogether novel suggestions were put into the form of resolutions, which were unanimously adopted. These are as follows:—

1. "That, as the nursing profession is closely linked as a subordinate profession with that of medicine, it should be dealt with as regards qualification of nurses in much the same way as medical practitioners; in other words, that a curriculum should be laid down for nurses and approved of by the Local Government Board, by the different medical and surgical licensing bodies, and have statutory authority, and that all nurses, having completed their training and possessing the necessary qualifications, should appear at certain specified dates for examination at different centres, before examiners appointed for the purpose, and the candidate, having passed such examination, should then have the right to have her name placed on the register of nurses."

2. "That we disapprove of any self-constituted bodies or boards being set up to deal with this question, and that Ireland should have power of registering its own nurses as it does its own doctors."

3. "That the Local Government Board for Ireland should determine by inspection whether or not nurses' training-schools and hospitals are sufficient for the purpose."

It will be seen that what is troubling both doctors and nurses is the possible disqualification of small hospitals, it having been pointed out by some of the speakers that a nurse could be quite as efficiently trained in a hospital with fifty beds as with three times the number. The suggestion is that the Local Government Board should arrange the curriculum, that the course of study and teaching should be left in the hands of the Irish medical profession, assisted by lady nurses, to be nominated in the first instance by the Government, and subsequently elected by the nurses on the Register. Resolution No. 2 shows that the Irish Nursing Board no more commends itself to the rural medical and nursing profession than the yet untried College of Nursing.

(Continued on page 538.)

### Nursing Affairs in Ireland.

(*concluded from p.* 532).

Lowering the Standard of Training.

Nurses should note that this scheme, like that of the Irish Nursing Board, contemplates nothing outside of registration, and that if it is adopted it must essentially tend to a lowering of the standard of nurses' training in Ireland. Small hospitals are, no doubt, most useful institutions in their way, but I should think perfectly hopeless as schools for training. Why not suggest that they should be made training-schools for students of medicine? Subject to their respective limitations the professions run hand in hand. Everybody in Ireland knows that in difficult cases the city physician or surgeon is sent for, or else, if it can be done, the patient is removed to the city hospital. It may be theoretically possible to have a first-class hospital limited to fifty patients, but in actual practice the average rural infirmary—a term which this Association proposes to drop—is necessarily lacking in modern equipment, and the variety of diseases treated there are at the lowest minimum. But this new proposition endorses my already expressed opinion that unrest is the predominant characteristic just now of the Irish nursing profession.

IERNE.

